# Multiple Intracranial Aneurysms Associated With Brain Arteriovenous Malformation: A Case Report and Treatment Strategies

**DOI:** 10.7759/cureus.59670

**Published:** 2024-05-05

**Authors:** Corneliu Toader, Luca-Andrei Glavan, Bogdan-Gabriel Bratu, Razvan-Adrian Covache-Busuioc, David-Ioan Dumitrascu, Alexandru Vladimir Ciurea

**Affiliations:** 1 Department of Neurosurgery, Carol Davila University of Medicine and Pharmacy, Bucharest, ROU; 2 Department of Neurosurgery, National Institute of Neurology and Neurovascular Diseases, Bucharest, ROU; 3 Department of Neurosurgery, Sanador Clinical Hospital, Bucharest, ROU

**Keywords:** endovascular coiling, surgical clipping, brain arteriovenous malformation, ophthalmic artery, anterior communicating artery, intracranial aneurysms

## Abstract

In the field of cerebrovascular neurosurgery, intracranial aneurysms (IAs) have been occasionally associated with brain arteriovenous malformations (BAVMs), indicating a more aggressive clinical course, and increased rates of hemorrhage and rehemorrhage. Treatment of flow-related IAs in BAVMs remains debatable, with considerations for preventive intervention versus concurrent BAVM treatment. Managing such situations might be challenging, especially in determining which of the IAs or BAVMs should be treated first, and which treatment strategy would be most appropriate for each situation. A precise identification of the rupture site is required, whether it is the AVM nidus or the IA, for choosing the best treatment plans. We present a case of a 29-year-old male patient diagnosed with several intracranial vascular conditions: a ruptured anterior communicating artery (ACoA) aneurysm and an unruptured ophthalmic artery aneurysm, associated with a frontal BAVM. Moreover, we discussed the possible scenarios regarding the association of these conditions, highlighting their manifestations and the most suitable therapeutic approach for each. Thus, our exploration of the challenges and considerations involved in treating these intricate neurovascular conditions underscores the need for a customized approach for each patient’s situation.

## Introduction

Intracranial aneurysms (IAs) represent a substantial area of interest and complexity in cerebrovascular neurosurgery, often appearing independently or in association with brain arteriovenous malformations (BAVMs). These aneurysms can be classified as proximal, intranidal, or distal flow-related when associated with BAVMs [1]. The occurrence of BAVMs in conjunction with aneurysms does not significantly vary based on gender, yet there is a tendency for females to have a higher incidence of hemorrhagic presentations. It is widely acknowledged that the coexistence of BAVMs and IAs correlates with increased rates of initial hemorrhage and rehemorrhage, indicating a more aggressive clinical course and natural history [2]. The reported prevalence of aneurysms associated with BAVMs varies significantly in literature, ranging from 2.7% to 58%, with most extensive case series suggesting a prevalence between 10% and 20% [3]. The annual risk of intracranial hemorrhage in patients with unruptured BAVMs and concurrent IAs is estimated at 7%, compared to 2%-4% for those with only BAVM [4].

The treatment approach for flow-related IAs in the context of BAVM is a topic of debate. While these aneurysms pose a higher risk for intracerebral hemorrhage (ICH), thus suggesting a more severe natural history, there is no universally accepted treatment strategy [5]. Treatment of proximal flow-related IAs is often preferred due to the risk of rupture during sudden hemodynamic changes at the time of BAVM resolution. Conversely, the potential for flow reduction through feeding arteries and subsequent regression of the IA post-BAVM extirpation has led to recommendations for addressing the BAVM initially [6]. Therefore, assessing the risk of flow-related IAs in patients with BAVM remains challenging, and no definitive guidelines exist. Moreover, it is unclear whether a more aggressive approach should be considered in high-risk patients with multiple aneurysms or a history of aneurysmal subarachnoid hemorrhage (aSAH).

## Case presentation

This case presents a 29-year-old male patient diagnosed with multiple intracranial vascular malformations. The patient presented to our clinic with cephalgia, nausea, and vomiting. A neurological exam revealed the patient was conscious, confused, with neck pain, intracranial hypertension syndrome, and Glasgow Coma Scale (GCS) = 14 points, corresponding to Grade 3 on the Hunt and Hess scale.

**Figure 1:**
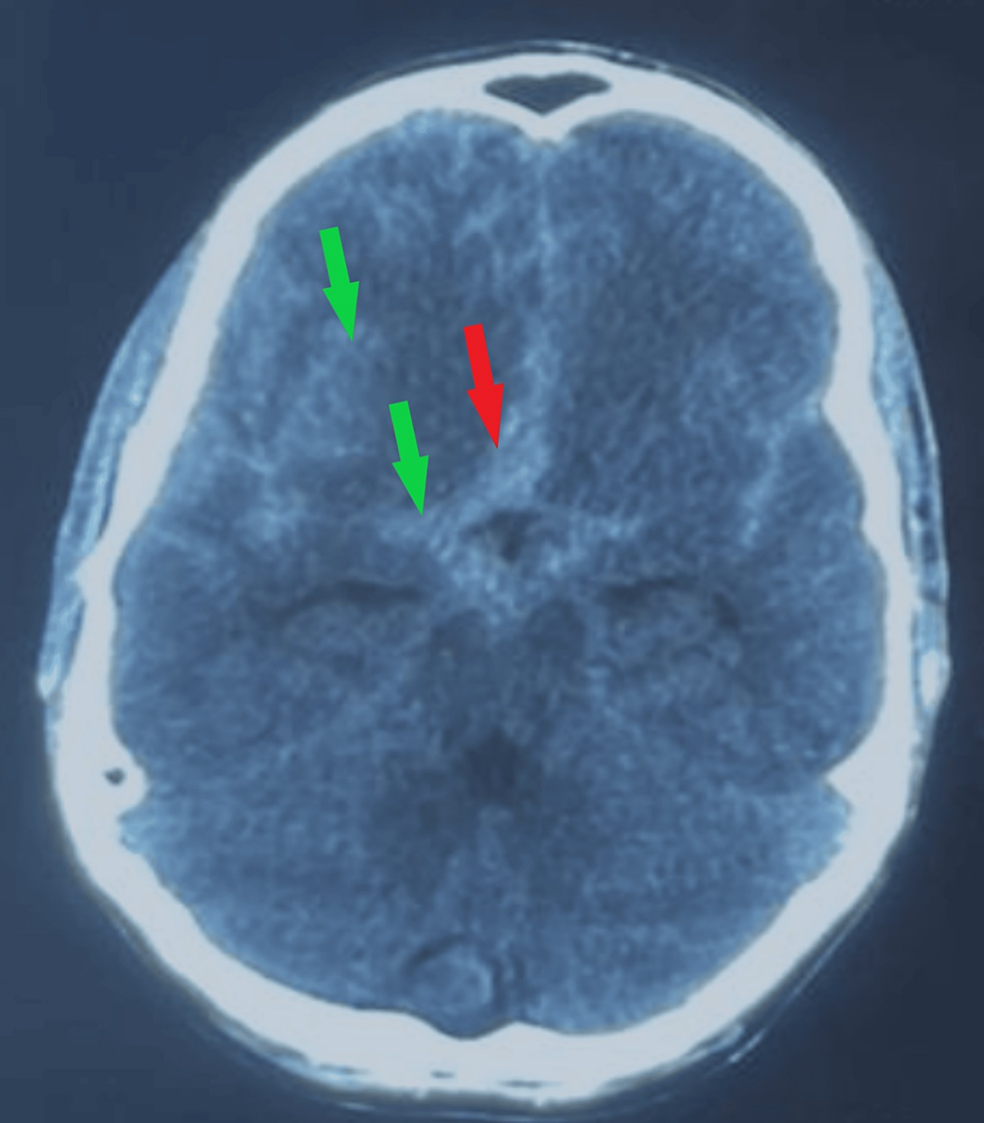
Preoperative CT scan. The image highlights a diffuse subarachnoid hemorrhage of more than 1 mm in thickness, with blood present within basal cisterns (red arrow) and right sylvian fissure (green arrows).

A CT scan was undergone, highlighting a subarachnoid hemorrhage Fisher Score 3, with associated cerebral edema (Figure 1).

**Figure 2:**
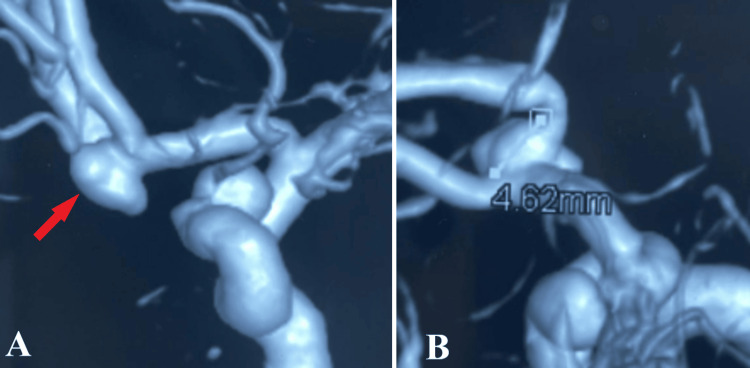
Preoperative 3D DSA. After 3D reconstruction, a high-quality aspect of the ACoA aneurysm (A, red arrow) is depicted, with aneurysm neck (B) measuring approximately 4.62 mm. 3D DSA: Three-dimensional digital subtraction angiography, ACoA: Anterior communicating artery.

To diagnose a possible intracranial vascular malformation, 3D digital subtraction angiography was performed, revealing a ruptured aneurysm of the anterior communicating artery with a maximum diameter of 7 mm and wide neck of 5 mm and a second unruptured aneurysm on the left ophthalmic segment of anterior cerebral aneurysm (Figure 2).

**Figure 3:**
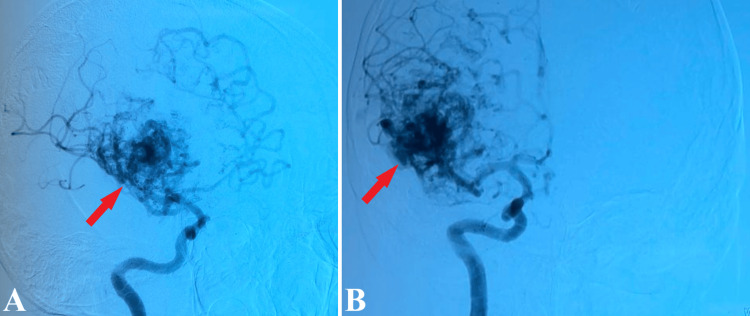
2D DSA of the arteriovenous malformation. 2D DSA, profile (A), and front (B) highlight the presence of a frontal arteriovenous malformation (red arrows). 2D DSA: Two-dimensional digital subtraction angiography.

Notable, a high flow right frontal arteriovenous malformation, Martin Spetzler grade III (medium-sized, 2 points and profound veins, 1 point), with nidus of approximately 5-6 cm, without visible intranidal changes, with multiple venous draining sources at the level of superior sagittal sinus and another vein through right transversal sinus (Figure 3).

Considering the patient's state due to lethal signs of subarachnoid hemorrhage, with significant Fisher Score 3 which might lead to vasospasm, the ruptured aneurysm had to be secured. After a left pterional flap, aneurysm clipping of the anterior communicating artery was achieved. Regarding the second aneurysm of the ophthalmic artery, the wrapping procedure was decided and performed during the clipping of the aforementioned aneurysm.

**Figure 4:**
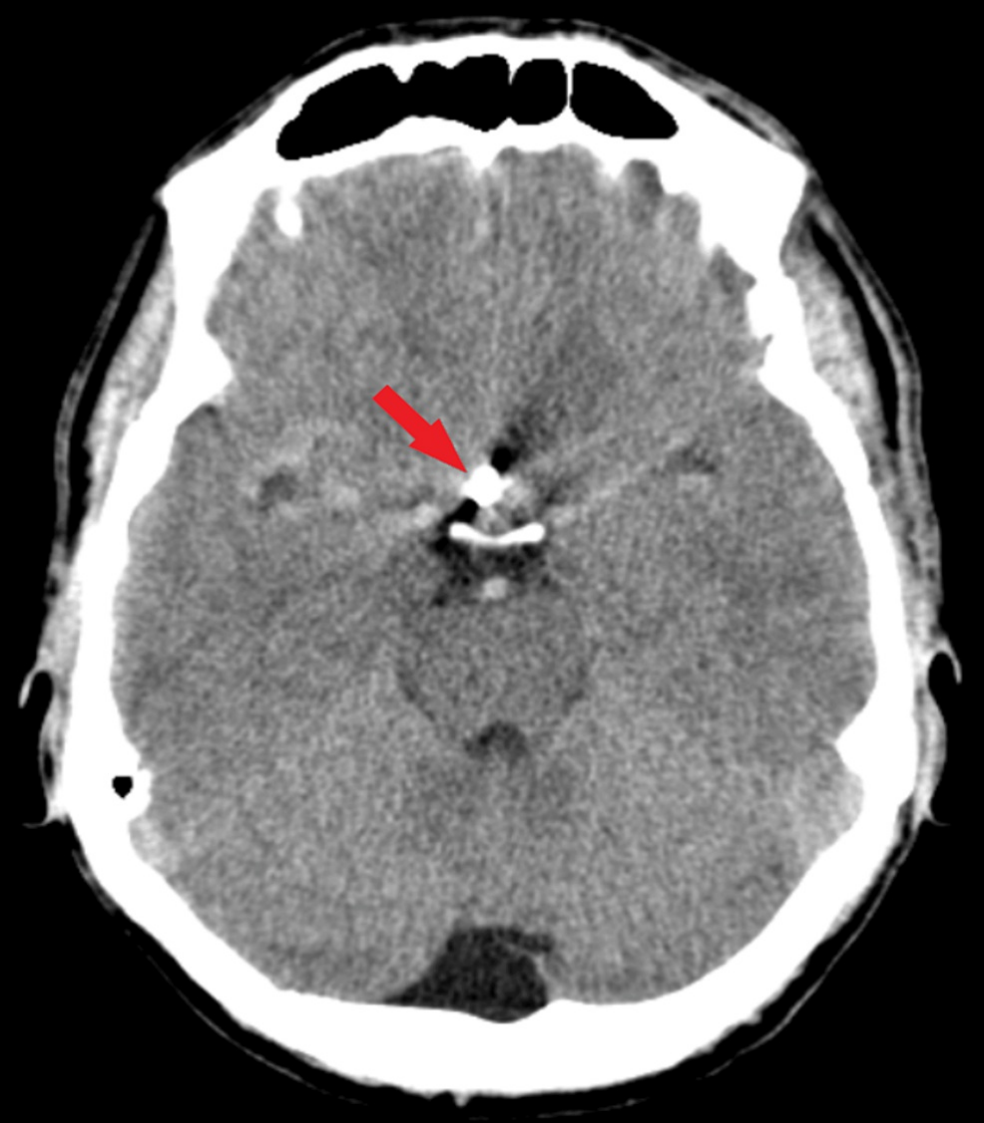
Postoperative CT scan. Axial section of CT scan tissular window demonstrate correct clip positioning (red arrow), without active hemorrhage.

Postoperatively, the patient showed important neurological improvements (modified Rankin Scale 1), and non-contrast CT scan findings revealed hyperdense areas according to the craniectomy, without mass effect to arteriovenous malformation and no signs of subarachnoid hemorrhage (Figure 4). At discharge, the patient was conscious, coherent, GCS = 15 points, with remitted intracranial hypertension syndrome, and no other neurological deficiencies were reported.

**Figure 5:**
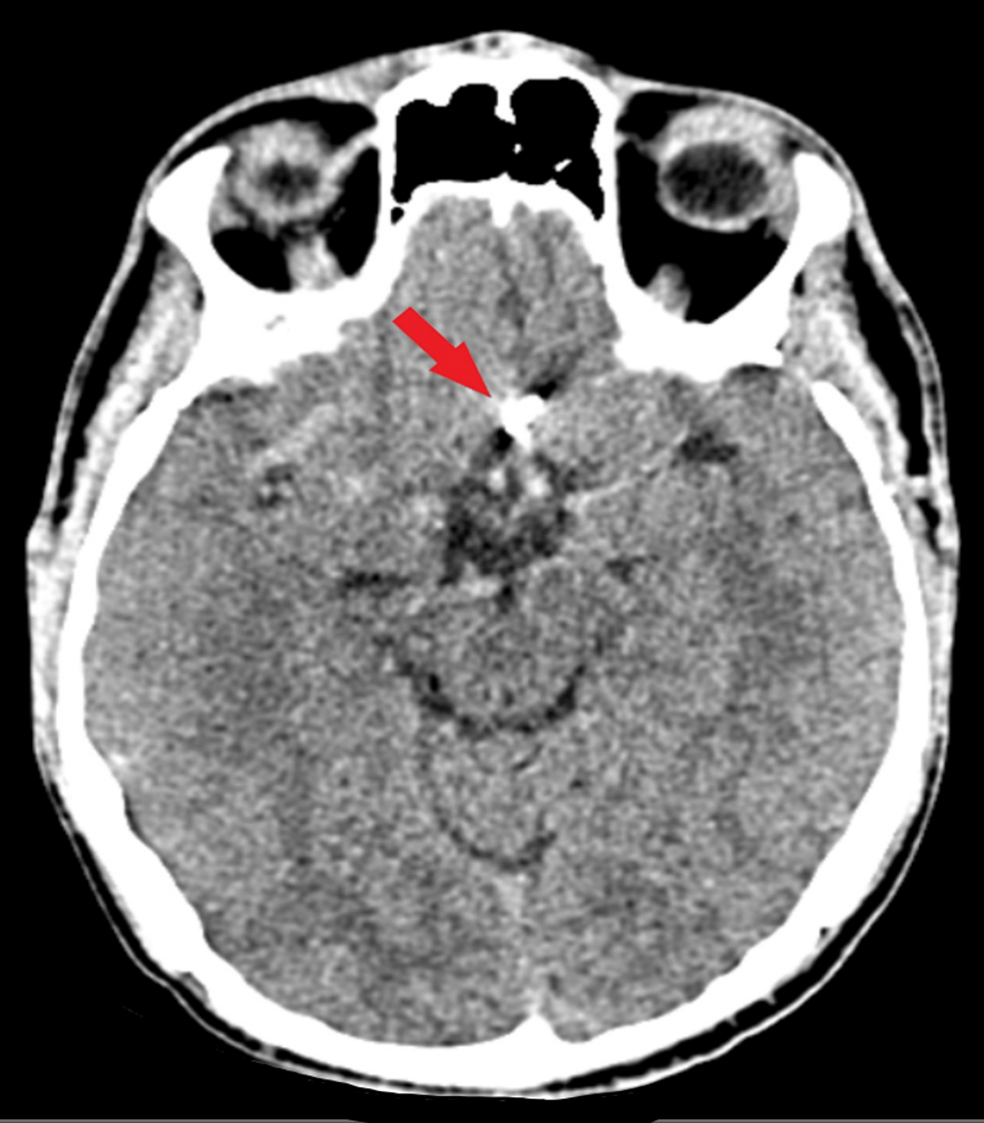
Follow-up CT scan. Axial section of a five-year follow-up CT examination, revealed the absence of an intracranial hemorrhage, with aneurysm clip present (red arrow).

Annually follow-ups for five years were registered, without new neurological deficits being observed, while neuroimaging scans showcased absence of subarachnoid hemorrhage and normal computer tomography aspect (Figure 5).

**Figure 6:**
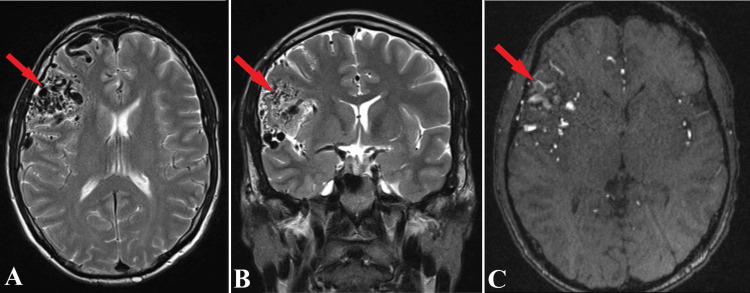
MRI scans of the arteriovenous malformation. Axial section of MRI T2 sequence (A), frontal section of MRI T2 sequence (B) and axial section of MRI T1 Gd sequence (C) offers a precise aspect of the frontal AVM (red arrows). Serpiginous trajectories are observed temporally and frontally at the level of the Sylvian fissure on the right side, suggesting the presence of vascular malformation. Spiral paths (C) capture the paramagnetic substance.

**Figure 7:**
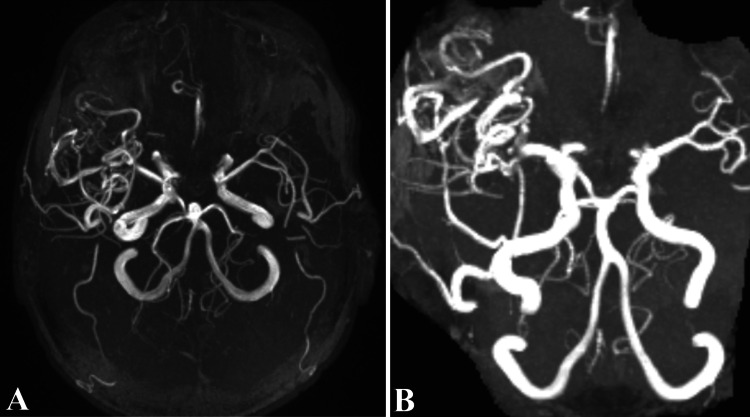
MR angiography of the arteriovenous malformation. Both images depict a 3D angiography perspective of the AVM through the MR angiography investigation, demonstrating draining sources into profound veins. We observe in the MR angiography image malformation of vascular trajectories starting in the M1 segment of the right middle cerebral artery and the posterior communicating artery on the right side. AVM: Arteriovenous malformation.

Additionally, MRI and MR angiography (Figures 6, 7) were undergone for an in depth understanding of the arteriovenous malformation aspect, remaining under observation. Moreover, considering the risk of AVM’s surgical resection, and patient’s young age, the right frontal arteriovenous malformation was left untreated.

## Discussion

Brain arteriovenous malformations represent a critical etiology of intracranial hemorrhage, predominantly in younger populations, and are correlated with elevated morbidity and mortality rates. The coexistence of BAVMs with intracranial aneurysms occurs at a frequency surpassing the expected rate of each pathology when considered separately. The identification rates of IAs in the context of AVMs have shown an increase, attributable to advancements in diagnostic methodologies, notably 3D and super-selective conventional angiography. Intracranial aneurysms associated with AVMs are implicated in an increased likelihood of hemorrhage upon initial presentation and a heightened risk of subsequent rehemorrhage, potentially leading to a more adverse natural history [7-10].

The presence of an aneurysm associated with a BAVM does not necessitate substantial alterations in the microsurgical techniques employed for the excision of the nidal component. Optimal exposure is essential. In scenarios where the treatment is oriented towards the IA instead of the BAVM, the surgical approach, including craniotomy, should be dictated by the aneurysm's location. Patients presenting with hemorrhage of indeterminate origin may benefit from an expansive surgical exposure, enabling the simultaneous and safe management of both pathologies and mitigating the risk of unforeseen and potentially critical intraoperative complications. The initial phase of resection involves the prompt application of clip ligation to all potential feeding arteries at the BAVM margin, succeeded by a detailed microsurgical dissection of the BAVM's periphery while conserving all feasible venous drainage. Early in the dissection process, a careful distinction between direct BAVM feeders and en passage vessels is imperative to avoid ischemia in brain regions distal to the nidus. This approach entails the selective targeting of small side branches feeding the BAVM while preserving the primary vessel. Distal flow-related aneurysms are infrequently associated with en passage vessels [2].

In the microsurgical management of brain arteriovenous malformations, small aneurysm clips are routinely utilized on direct feeders exceeding a few millimeters in luminal diameter. This clipping strategy is particularly effective for handling fragile, enlarged periependymal arterioles, which often resist bipolar cauterization. Despite the meticulous and systematic circumferential nidal dissection, this microsurgical technique limits the surgeon’s dissection to the gliotic margins of the BAVM. Operating at the interface of abnormal gliotic brain tissue and functional parenchyma, the surgeon employs tools such as bipolar cautery, microscissors, aneurysm clips, and a constantly present suction tip for gentle retraction. This approach is designed to minimize iatrogenic damage to the surrounding normal brain tissue and crucial adjacent vasculature [11]. However, it does not completely eliminate the risk of accidentally isolating small portions of the malformation, a complication that may lead to serious intraoperative or postoperative hemorrhage and potentially severe neurological outcomes.

Conversely, recent studies have investigated the efficacy of endovascular neurointerventional techniques in treating BAVMs. Advanced endovascular tools, such as liquid embolic agents (e.g., Onyx and N-butyl cyanoacrylate) and flow-directed or flow-assisted microcatheters, have significantly expanded treatment options. Detachable coils are employed for embolizing high-flow arteries, directly treating aneurysms, or selectively reducing flow to enable safer use of liquid embolic agents without risking venous system shunting. The primary advantage of endovascular management lies in its role as an adjunct to microsurgical and radio-surgical techniques, forming a multimodal approach that has become standard care for certain BAVM subtypes. Preoperative embolization has been shown to be beneficial, often essential, for reducing BAVM flow prior to surgery and limiting intraoperative blood loss and operating time. However, the overall effectiveness of this strategy remains a topic of debate. Preoperative embolization continues to be the predominant approach in most neurosurgical centers. Recent reports suggest that early embolization of ruptured BAVMs can be safe, especially when targeting high-risk features to reduce rehemorrhage rates [12].

In the International Subarachnoid Aneurysm Trial (ISAT), researchers conducted the first multicenter, randomized trial to assess the safety and efficacy of endovascular embolization versus microsurgical clipping. This trial, involving 42 centers across Europe and North America, enrolled 2143 patients, who were randomly allocated to either neurosurgical clipping (n = 1070) or endovascular embolization using bare platinum coils (n = 1073). The primary endpoint was the proportion of patients with a modified Rankin Scale score of 3 to 6 (indicating dependency or death) at one year. The trial was prematurely terminated by the steering committee following an interim analysis that demonstrated inferior outcomes in the neurosurgical clipping group. Specifically, 190 of 801 (23.7%) patients in the endovascular group were either dependent or had died at one year, compared to 243 of 793 (30.6%) in the microsurgical clipping group (P = 0.0019). This translated to relative and absolute risk reductions in dependency or death of 22.6% (95% CI, 8.9 to 34.2) and 6.9% (2.5 to 11.3), respectively, favoring endovascular treatment [13].

Subsequent to the early termination of ISAT enrollment, the investigators continued to monitor the long-term outcomes of the enrolled patients. The data indicated that the initial survival benefit observed in the endovascular group persisted for up to seven years and remained statistically significant (log-rank, P = 0.03) [14]. The absolute risk reduction in death or dependency in the endovascular cohort increased marginally to 7.4%. Additionally, the incidence of epilepsy was notably lower in patients who underwent coil embolization. However, there was a marginally increased risk of rebleeding at the seven-year follow-up in the endovascular group, recorded at 0.2% per patient-year, compared to 0.1% per patient-year in the surgical clipping group (log-rank, P = 0.22) [14].

The Cerebral Aneurysmal Rupture After Treatment (CARAT) study focused on comparing rerupture rates following aneurysmal subarachnoid hemorrhage [15]. This investigation found that, over one year, rerupture of the treated aneurysm occurred in one patient who underwent coil embolization during 904 person-years of follow-up (an annual rate of 0.11%), whereas no reruptures were observed among patients treated with surgical clipping over 2666 person-years (P = 0.11). Additionally, aneurysm retreatment after one year was more frequent in patients who received coil embolization, though major complications during retreatment were rare. The study concluded that late events are unlikely to significantly alter the differences observed between the procedures at the one-year follow-up [15].

Regarding the treatment of flow-related intracranial aneurysms associated with brain arteriovenous malformations, intervention might be indicated based on the potential for rupture prior to BAVM elimination. Considering a history of aSAH, a preference towards direct endovascular coil embolization is often argued [16,17]. However, the use of adjunctive devices for assisted coiling, such as flow diversion, may not be suitable due to the required use of antiplatelet agents and the pending treatment of the BAVM. The impact of BAVM treatment on the natural history of proximal flow-related IAs is not extensively documented in the literature. Eliava et al. reported a case where an aneurysm spontaneously regressed following AVM treatment among a total of 205 aneurysms associated with BAVM [18].

In treating IAs associated with AVMs, it is crucial to determine the precise rupture site in cases of hemorrhagic presentation. The anatomical relationship between the IAs and the AVM nidus must be thoroughly assessed when formulating treatment plans. Identifying the hemorrhage source-whether it's the AVM nidus or the IA-is vital, with the diagnosis relying on clinical experience and inference. This determination becomes more evident when the hemorrhage location on the head CT scan obtained at presentation is spatially distant from the nidus [19]. Correlation with angiographic studies, including superselective injections, further assists in this assessment. Subarachnoid hemorrhage without accompanying intracerebral hemorrhage typically indicates the IA as the likely bleed source. Likewise, a focal hematoma adjacent to the IA, extending secondarily to the subarachnoid space, suggests the IA as the rupture origin [20].

If the intracranial aneurysm is identified as the source of hemorrhage, it should be addressed as promptly as feasible, following the same treatment guidelines applicable to isolated saccular arterial aneurysms. In situations where the aneurysm is proximal to the arteriovenous malformation and the AVM is amenable to surgical resection, both lesions ought to be managed in a single operative session. Proximal flow-related aneurysms require treatment through surgical or endovascular methods, contingent on their location, morphology, and the experience of the operator [2,9]. The intervention for the associated AVM might be deferred and can encompass surgical, endovascular, radio-surgical, or conservative approaches. In cases where an associated aneurysm is the source of bleeding and AVM treatment is not indicated, the aneurysm should be addressed either endovascularly or surgically. Nevertheless, a notable recurrence rate of aneurysms has been observed following endovascular treatment in the absence of definitive nidal obliteration [9]. Yu et al. conducted a study involving 16 aneurysms, of which only two were unruptured [21]. These aneurysms were treated first, followed by the treatment of AVMs using an embolization approach. The patients' uneventful follow-up outcomes implied that addressing ruptured aneurysms initially may enhance the overall treatment results [21].

When the hemorrhage source is a pseudoaneurysm originating from a small perforating branch, immediate treatment or close imaging surveillance is necessary. Pseudoaneurysms, lacking a true wall, typically exhibit a dynamic behavior in the acute phase, characterized by early expansion and rerupture, or even spontaneous regression. If a pseudoaneurysm is treated, the AVM can be addressed electively at a later time, as the hemorrhage source has been secured. Endovascular techniques are particularly beneficial for pseudoaneurysm treatment, especially when intravascular access near the pseudoaneurysm is feasible, considering most pseudoaneurysms are situated on perforating arteries that are challenging to access surgically [20].

When the source of hemorrhage is identified as originating from an arteriovenous malformation or an intranidal aneurysm, urgent treatment may not be necessary, as the risk of early rerupture is generally low, barring the presence of impaired venous outflow from the nidus. Initially, a conservative management approach can be adopted, with an angiogram scheduled for 4-6 weeks later. Subsequently, if the risk-benefit assessment of any proposed intervention versus the natural history of the lesion is favorable, elective management of the AVM and the intranidal aneurysm can be pursued. Preoperative embolization targeting high-flow fistulas and associated prenidal and intranidal intracranial aneurysms before definitive surgery is a viable strategy, although its efficacy remains to be conclusively established. Currently, it is unclear whether palliative embolization of the nidus or feeding pedicle of the AVM, particularly when addressing an intranidal aneurysm, effectively reduces the risk of recurrent hemorrhage [22].

In cases of intracerebral hemorrhage, the brain arteriovenous malformation or BAVM-nidal aneurysm is typically implicated as the cause. The likelihood of early rehemorrhage in these instances is relatively low. When the BAVM is determined to be the hemorrhage source and there is no indication for the surgical removal of an intraparenchymal hematoma, the patient should receive medical management during the acute phase. A follow-up catheter angiography is advised in four to six weeks, post-hematoma reabsorption. Subsequent treatment planning should be meticulous, aiming to obliterate both the BAVM and IA if feasible. If the source of bleeding cannot be definitively determined by the treating neurosurgeon, both lesions should ideally be treated concurrently, if possible. However, if simultaneous treatment is not feasible, priority should be given to treating the aneurysm first due to its higher rerupture risk, which contributes to increased morbidity and mortality [23].

## Conclusions

Brain arteriovenous malformations (BAVMs) have long been acknowledged as formidable challenges in the realm of neurovascular pathologies. Recent studies have further highlighted the complexity of these conditions by demonstrating their frequent association with intracranial aneurysms (IAs), adding layers of difficulty to their management. Despite ongoing research and clinical experience, there remains a lack of consensus on the optimal treatment strategy for BAVMs and associated IAs. The inherent risks associated with microsurgical and endovascular interventions must be carefully balanced against the natural progression of the disease, which is not yet fully understood.

In exploring this patient's case, we have navigated the intricate and multifaceted nature of managing BAVMs in conjunction with IAs; our 29-year-old patient was diagnosed with two IAs, alongside a right frontal BAVM. The symptoms suggested a subarachnoid hemorrhage, attributable to these aneurysms. Considering the severity of the condition (Fisher Score 3, thus a high probability of a fatal new rupture) and the intricate risk of surgical resection for the BAVM, both aneurysms were addressed simultaneously, while the right BAVM was left untreated.

Our research and clinical observations have revealed several key insights into the complexities inherent in such cases. This work, therefore, stands as a significant contribution to the body of knowledge on this topic, offering a detailed exploration of the challenges and considerations involved in treating these intricate neurovascular conditions.
